# Analysis of Car-Following Behaviors under Different Conditions on the Entrance Section of Cross-River and Cross-Sea Tunnels: A Case Study of Shanghai Yangtze River Tunnel

**DOI:** 10.3390/ijerph191911975

**Published:** 2022-09-22

**Authors:** Ting Zhang, Feng Chen, Yadi Huang, Mingtao Song, Xiao Hu

**Affiliations:** 1 The Key Laboratory of Road and Traffic Engineering, Ministry of Education Tongji University, Shanghai 201804, China; 2 China Academy of Urban Planning and Design, Western Branch, Chongqing 400000, China; 3 Traffic Science and Technology of Guangdong Hualu Limited Company, Guangzhou 510420, China; 4 Shanghai Urban Operation (Group) Co., Ltd., Shanghai 200023, China

**Keywords:** traffic safety, car-following behavior, entrance section of cross-river and cross-sea tunnels

## Abstract

Compared to highway road tunnels, the entrance section of cross-river and cross-sea tunnels feature long and steep slopes. Along with a complicated traffic environment and harmful weather conditions, traffic congestion and rear-end crashes occur frequently during car-following in cross-river and cross-sea tunnels. It is necessary to examine the impact of traffic flow and weather conditions on car-following behavior at the entrance section of cross-river and cross-sea tunnels. To this end, this paper first extracted the vehicle speed data based surveillance video at the entrance of the Shanghai Yangtze River Tunnel. Moreover, the actual average speed under different traffic flow conditions was obtained through the clustering algorithm, which was used as the basis for setting the experimental parameters. Then, in the driving simulation experiment, three traffic flow conditions (free flow, congested flow, and jam flow) were set up in three weather conditions (sunny, rainy, and snowy), and a risk situation was set up in each condition. Distance headway, time headway, acceleration, lateral offset, and driver’s emergency response time were collected. Moreover, seven slopes of 2% to 5% were set, and the relationship of slope on longitudinal speed and lateral offset was analyzed. ANOVA and post-hoc analyses were applied. The result indicates that traffic flow conditions have a significant effect on the car-following behavior, while weather conditions mainly influence the time headway. Moreover, drivers tend to adopt more cautious driving behavior as the distance between the vehicle and the tunnel entrance decreases. The results also show that the slope of the cross-river and cross-sea tunnel entrance section has a major influence on vehicle speed.

## 1. Introduction

Due to serious accident consequences and economic losses, traffic congestion and traffic accidents in tunnels have been major problems for transportation management agencies. In recent years, cross-river and cross-sea tunnels have developed rapidly, like most urban underground roads and road tunnels. A long and steep slope is generally set at the entrance sections of cross-river and cross-sea tunnels to create a connection between the river and the sea. This feature is a unique feature that distinguishes cross-river and cross-sea tunnels from common road tunnels. Moreover, this trait has been shown to influence driving behavior and be positively correlated with accident frequency in cross-river and cross-sea tunnels, especially in the entrance section [1]. Along with traffic flow and weather conditions, the long and steep slope might further complicate driving safety in the entrance section of the cross-river and cross-sea tunnel.

There have been many research attempts to understand the potential relationship between the tunnel environment and driving behavior. Previous studies found that the accident probability in the tunnel section is higher than in other road sections [2,3]; the accident probability of the tunnel entrance section is higher than that of the tunnel exit section [4]. Due to the dramatic changes in light, landscape, and other conditions at the entrance section of the tunnel, driving behavior at the entrance section of the tunnel may change. Shao et al. [5] found that slope had a significant effect on driving performance. Besides, it was shown that rear-end collision risk was higher under the long and steep slope. Akamatsu et al. [6] evaluated the effects of the tunnel environment on the driver’s nervousness (measured as accelerator pedal force) and found that the speed change was particularly noticeable 50 m before entering the tunnel. Wang et al. [7] observed that drivers started decelerating about 100 m before entering the tunnel and ended the process approximately 50 m after entering the tunnel; drivers began to decelerate about 100 m before the tunnel exit and ended the process immediately upon exiting the tunnel. Wan et al. [8] conducted a quantitative analysis of the influence of tunnel brightness on the driving speed of drivers, concluding that the greater the tunnel brightness value, the more precise the driver’s perception of the vehicle’s speed would be and the faster the reaction time would be.

In addition to the above research, some researchers have also attempted to examine the impact of different conditions on car-following behavior. Shi et al. [9] analyzed the difference between driving behaviors under sunny and hazy weather conditions. It was shown that in lower visibility conditions, the average value and variance of driver acceleration and deceleration behavior variables and expected speed were smaller, indicating that drivers drove more cautiously in low visibility conditions. Using driving simulation experiments, Kang et al. [10] investigated the changes in headway, headway variance, and other indicators of car-following behavior in various weather conditions. It appears that drivers were more difficult to respond to changes in lead vehicle speed than to changes in headway. Ibrahim et al. [11] used the dummy variable regression analysis method on the traffic data in snowy weather, it is concluded that the vehicle speed was reduced by 3% to 5% in light snow weather and by 30% to 40% in heavy snow weather. During low visibility weather conditions, Rosey et al. [12] studied changes in headway and the results showed that when the visibility is 150 m, the proportion of vehicles with a headway less than 2 s is 2.5 times that of normal traffic flow under normal conditions. Minderhoud et al. [13] found that during traffic shocks, the time headway is positively correlated with the speed, while the time headway is not correlated with the speed, and drivers are likely to maintain a constant headway during the following. Piao et al. [14] used an experimental car to obtain data on drivers’ car-following behavior in three European cities. Data analysis revealed a positive correlation between the car-following distance and speed. Moreover, compared with a low-speed car-following method, the fluctuation in the car-following distance is also larger when a high-speed car-following method is used.

In general, driving behavior in tunnels has been analyzed from a variety of perspectives. However, as for the long slope section of cross-river and cross-sea tunnels, the driver’s stress and anxiety could be enhanced when a variety of factors are influenced at the same time. Besides, the driving environment and traffic flow characteristics of the entrance section of the cross-river and cross-sea tunnel are quite different from other tunnels and highways. The characteristics of car-following behavior might be quite different from the common road tunnel. Therefore, in order to study the car-following behavior characteristics of the entrance section of the cross-river and cross-sea tunnel under various conditions, according to the traffic environment characteristics of the cross-river and cross-sea tunnel, this paper selected the entrance section of the Shanghai Yangtze River Tunnel as the typical entrance section of the cross-river and cross-sea tunnel. Using a driving simulation experiment, this paper investigated the differences in the car-following behavior characteristics at the entrance section of a cross-river and cross-sea tunnel under various conditions.

## 2. Methods

### 2.1. Apparatus

**Figure 1:**
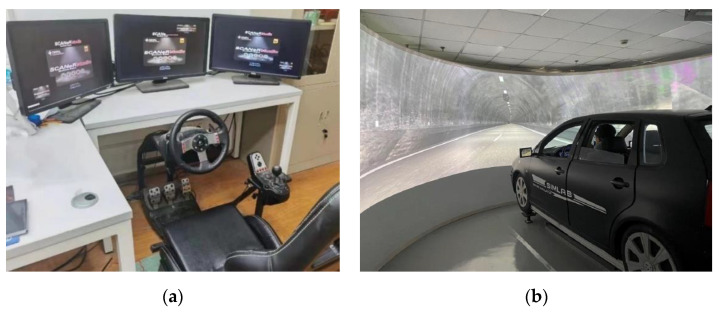
Driving Simulation Experimental Apparatus. (**a**) Driving simulator used in car following experiment. (**b**) 3-degree of freedom motion-based driving simulator.

The simulation software used SCANeR studio 1.6 in the car-following experiment. The simulation software can be used for 3D road design, landscape design, and vehicle dynamics model construction. In addition, the software can adjust the visibility, wind speed, road adhesion coefficient, weather conditions, and other experimental conditions, providing comprehensive technical support for experimental design and data analysis. The driving simulator included three LED displays, a driving seat, an accelerator pedal, a brake pedal, a steering wheel, a gear shift lever, etc. A visual system provided a 135° (horizontal) forward view of the simulated roadway (see Figure 1a).

The experiment with different slopes was conducted on a 3-degree of freedom motion-based driving simulator (see Figure 1b). It is run with SILAB^®^ 6.0 (WIVW GmbH, Veitshöchheim, Germany). Its degrees of freedom can give the driver a more realistic perception of the slope.

During the experiment, the driving simulator recorded a lot of data, such as time, trip meter, driving speed, lane deviation, acceleration, etc.

### 2.2. Scenarios

**Figure 2:**
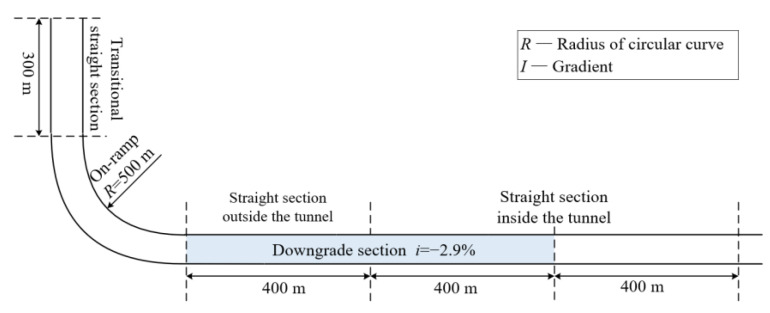
Schematic diagram of experiment scene.

This study was based on the real geometry of the entrance section of the Shanghai Yangtze River Tunnel and Bridge. It was 2.6 km in length with a speed limit of 80 km per hour. The lane widths, longitudinal gradients, sight distances, and other geometrical characteristics were incorporated into the simulation (see Figure 2).

**Figure 3:**
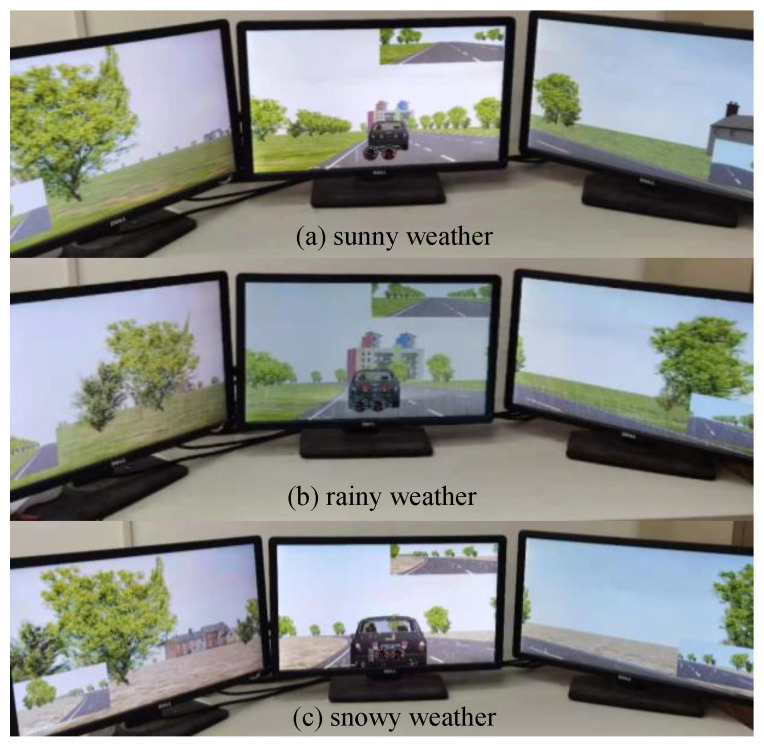
The driver’s vision.

The scenarios were created to simulate different weather conditions for drivers, including sunny weather, rainy weather, and snowy weather (see Figure 3). For all weather conditions, the scenarios were set up to simulate three conditions of traffic flow: free flow, congested flow, and jam flow. In total, nine scenarios were built up, as shown in Table 1:

Considering the negative impact of adverse weather conditions on visibility, and the road adhesion coefficient, it was necessary to set up different weather conditions and their corresponding adhesion coefficients to investigate the effect of weather-adhesion coefficient on driving behavior. Different scenarios were created to simulate different weather conditions for drivers, including sunny weather, rainy weather, and snowy weather. In the experiment, the road adhesion coefficient is adjusted according to the weather conditions in the driving simulator. Three weather conditions and parameters of the tunnel’s adhesion coefficient were selected [15], as shown in Table 2.

In order to reflect the traffic condition of the entrance section of the cross-river and cross-sea tunnel, the video surveillance at 50 m within the entrance of the Shanghai Yangtze River Tunnel on 1–8 October 2018 was observed, and the video at 11:00–12:00 on 1 October 2018 was selected. During this period, traffic conditions were congested flow and jam flow, and there was almost no free flow. Video processing software Adobe Premiere Pro CC 2018 was used to extract data from the video. The speed data of 1351 passenger cars in the inner lane of the tunnel entrance were counted. Then the vehicle speed values in the congestion flow and the jam flow are divided by using the K-means clustering algorithm. The speed clustering results are shown in Table 3. Using the clustering results, the speed of the preceding vehicle was determined under three traffic flow conditions: free flow—80 km/h, congested flow—40 km/h, and jam flow—20 km/h.

### 2.3. Data Collection Procedure

Before the driving simulator experiment, participants were requested to fill in a questionnaire concerning their basic information (e.g., gender, age, driving years). Next, the participants were asked to drive approximately five minutes in an environment unrelated to the experiment in order to become familiar with the operation of starting, accelerating, decelerating, changing lanes, and turning off the driving simulator.

(1) The experiment with different weather and traffic conditions

**Figure 4:**
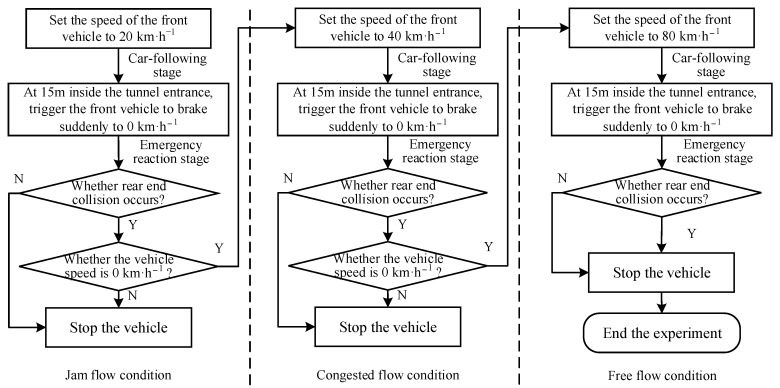
The experimental process flow diagram.

There were nine driving scenarios in the car-following experiment. The lead vehicle was programmed to travel on the lane throughout the simulation of the scenario. To study the driver’s emergency reaction, a sudden braking event of the preceding vehicle was set up in all scenarios. The experimental process program is shown in Figure 4. Participants were not informed about traffic conditions before the experiment. A short break of a few minutes was taken between every two scenarios based on the needs of the participants.

(2) The experiment with different longitudinal slopes

Through an investigation on the alignment of cross-river and cross-sea tunnels in China, the longitudinal slope (first 400 m of the entrance section of cross-river and cross-sea tunnel) was varied on seven levels, which were 2.0%, 2.5%, 3.0%, 3.5%, 4.0%, 4.5% and 5.0%. The sequence of the seven scenarios was randomly assigned. They were also informed to drive as they usually would with an 80 km/h speed limit and they were allowed to take a break between scenarios on demand. 

The collected data from the driving simulator were recorded at a frequency of 10 Hz. More specifically, the variables were collected to reveal driving behavior, including the speed, maximum acceleration, and deceleration rates, mean acceleration and deceleration rates, maximum and mean throttle force, and maximum and mean brake pedal force. Table 4 provides the definitions of these variables.

### 2.4. Participants

In the experiment with different weather and traffic conditions, 33 participants (24 males and nine females) were recruited from a population of licensed drivers who had at least two years of driving experience. The average driving experience was 5.67 years, with a standard deviation of 4.26 years. The participants were aged between 22 and 45 years old (mean = 27.4 years, standard deviation = 5.84 years). 

In the experiment with different longitudinal slopes, 32 participants (26 males and six females) were recruited from a population of licensed drivers who had at least two years of driving experience. The average driving experience was 6.22 years, with a standard deviation of 4.58 years. The participants were aged between 22 and 49 years old (mean = 28.4 years, standard deviation = 6.55 years). 

Their corrected vision was above 5.0. None of the participants showed symptoms of simulator sickness and all finished the test drive.

### 2.5. Car-Following Behavior Variable

(1) Distance headway

The distance headway refers to the distance between the same ends of the adjacent vehicles (front or rear) driving in the same lane and in the same direction. Relative to the speed of the preceding vehicle, it is easier for the driver of the following vehicle to judge the distance headway [10]. According to “Chinese Expressway Traffic Management Measures” [16] stipulates: For vehicles driving on the expressway, the actual speed of the vehicle is as many kilometers per hour, and the distance between the vehicle and the vehicle in front should be kept at least as many meters, that is, if the driving speed is 100 km/h, the distance between vehicles should be kept at more than 100 m; if the driving speed is 70 km/h, the distance between vehicles should be kept at more than 70 m; in case of fog, rain or snow, they should slow down. In this experiment, the safe vehicle distance should be maintained at 20 m, 40 m, and 80 m in both jammed and congested conditions.

(2) Time headway

The time headway describes the time interval between two consecutive vehicles in the same lane passing through the same road cross-section and represents the maximum reaction time of the driver of the following vehicle when the front vehicle brakes. The time headway is an important indicator of the spatiotemporal characteristics of the car-following process. Each driver has a preferred time headway when following the vehicle. The driving difficulty and psychological load will increase if the actual time headway is less than the preferred time headway.

(3) Acceleration

The acceleration of a vehicle refers to the degree of change in speed per unit time, which directly influences the change in speed. In this study, the acceleration value is positive, indicating the vehicle is accelerating; the acceleration value is negative, indicating the vehicle is decelerating.

(4) Lateral offset

**Figure 5:**
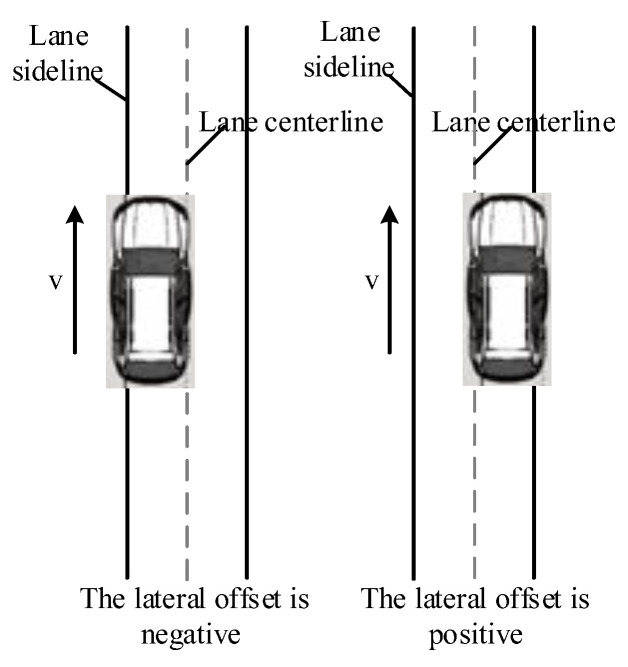
The meaning of positive and negative values of lateral offset.

Lateral offset is the distance between the centerline of the vehicle and the centerline of the lane, which describes the lateral trajectory of the vehicle and the driver’s lateral control of the vehicle. As shown in Figure 5, when the vehicle centerline is on the left side of the lane centerline, the lateral offset is negative, otherwise, the lateral offset is positive.

(5) Driver’s emergency response

Due to the difference in the driver’s judgment stage and reaction stage, to better quantify and reflect the driver’s reaction speed in the different conditions, an auxiliary reference coefficient of “time to half speed” was introduced in this study, which is the time that it takes for the vehicle to drop from its initial speed to half of the speed [17].

## 3. Data Analysis and Results

### 3.1. The Influence of Different Conditions on Distance Headways

**Figure 6:**
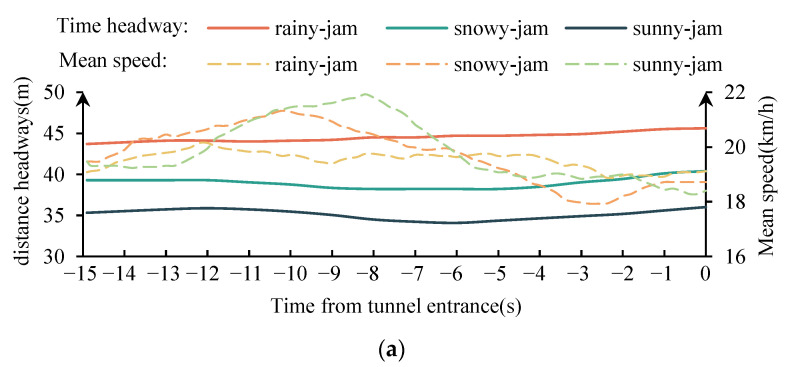

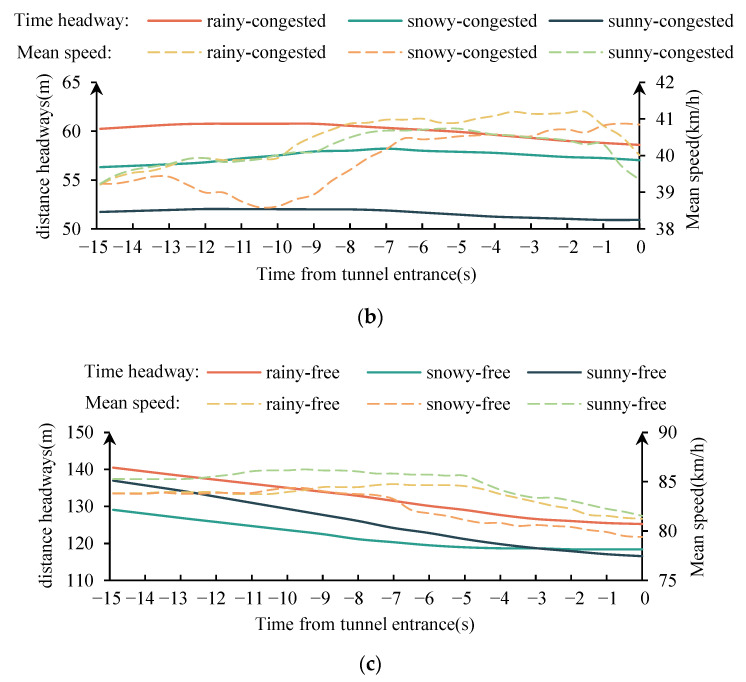
The average change of the distance headways within 15 s from the tunnel entrance. (**a**) jam flow condition. (**b**) congested flow condition. (**c**) free flow condition.

As shown in Figure 6, the average distance headways in this experiment met the requirements for a safe distance. Under jam and congested flow conditions, the values of distance headways in different weather conditions were rainy weather > snowy weather > sunny weather. The reason was that in rainy and snowy weather, the driver’s field of vision was not as good as in sunny weather. Therefore, they tended to keep a larger distance headway and follow carefully. Moreover, the range of the distance headway was maintained between 35 and 45 m and 50 and 60 m in jam flow and congested flow, which did not change with the distance from the tunnel entrance and remained relatively constant. Under free flow conditions, the effect of different weather conditions on distance headway was small; however, the distance headway was larger than under jam flow and congested flow conditions, averaging between 120 and 140 m. In addition, the closer the distance was to the tunnel entrance, the shorter the distance headway tended to be.

ANOVA should meet the requirements of normality and variance homogeneity test. The assumption of normality was checked by a Q-Q plot. The results showed no clear sign of violation. The Levene test was used to determine whether the variance met the requirements for homogeneity. The one-way ANOVA was used to assess the significance of mean differences between groups if the variance was homogeneous (*p* > 0.1), while the LSD test was employed to assess the significance of pairwise differences between groups if the variance was uneven (*p* > 0.1); in the case of uneven variance (*p* > 0.1), the Welch test was administered to assess the significance of mean differences, while the Games–Howell test was employed to assess the significance of pairwise differences between groups.

The minimum distance headway is the minimum safe distance that the driver can accept in the process of car-following, which can most intuitively describe when the driver is exposed to the greatest risk during the process. The results of two-way ANOVA showed that traffic flow (*p* = 0.000 < 0.05) had a significant impact on the minimum distance headway, whereas weather (*p* = 0.266 > 0.1) and the interaction between weather and traffic flow (*p* = 0.940 > 0.1) had no significant impact on the minimum distance headway. A further study was conducted on the influence of traffic flow on the minimum distance headway, and the results are shown in Table 5.

**Figure 7:**
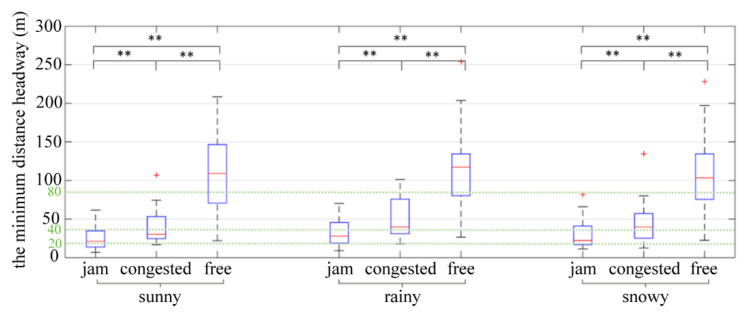
The minimum distance headway. ** indicates the variable was statistically significant at 0.05 level. + indicates outlier.

As shown in Figure 7, under different weather conditions, the minimum distance headway followed the law of free flow > crowded flow > crowded flow, and the mean difference is significant. The median of the minimum distance headway met the requirements of the safe distance, but the lower quartiles were all lower than the safe distance required by “Chinese Expressway Traffic Management Measures”. Specifically, under the congested flow condition, more than 50% of drivers’ minimum distance was below the safe distance. The distance headway was too close if the vehicle in front broke or changed lanes suddenly, the driver would have little time to react, which often resulted in traffic accidents.

As the distance headway differs from the driver’s expectation, the rear driver adjusts the speed in order to maintain the desired distance headway. The change in vehicle speed in traffic flow is one of the factors affecting the stability of traffic flow [18]. The standard deviation of the distance headway indicates the degree of fluctuation of the distance headway during the car-following process, which indirectly indicates how stable the driving process is. The results of a two-way ANOVA showed that traffic flow (*p* = 0.000 < 0.05) had a significant impact on the standard deviation of distance headway, whereas weather (*p* = 0.640 > 0.1) and the interaction between weather and traffic flow (*p* = 0.806 > 0.1) did not significantly impact the standard deviation of distance headway.

**Figure 8:**
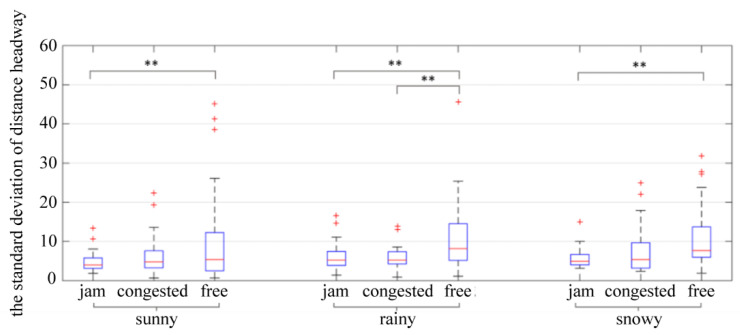
The standard deviation of distance headway. ** indicates the variable was statistically significant at 0.05 level. + indicates outlier.

Further study of the impact of traffic flow on the standard deviation of distance headway is shown in Table 6 and Figure 8. It can be seen that under different weather conditions, the standard deviation of distance headway shows the law of free flow > congested flow and jam flow. In the above analysis, the distance headway did not vary with the distance from the tunnel entrance in congested flow conditions or jam flow conditions, and it was relatively constant. In the free flow condition, the distance headway was relatively larger, and as the tunnel entrance approached, the distance headway decreased. Thus, the standard deviation of the distance headway in the free flow condition was higher than that in the congested flow and jam flow conditions.

### 3.2. The Influence of Different Conditions on Time Headways

**Figure 9:**
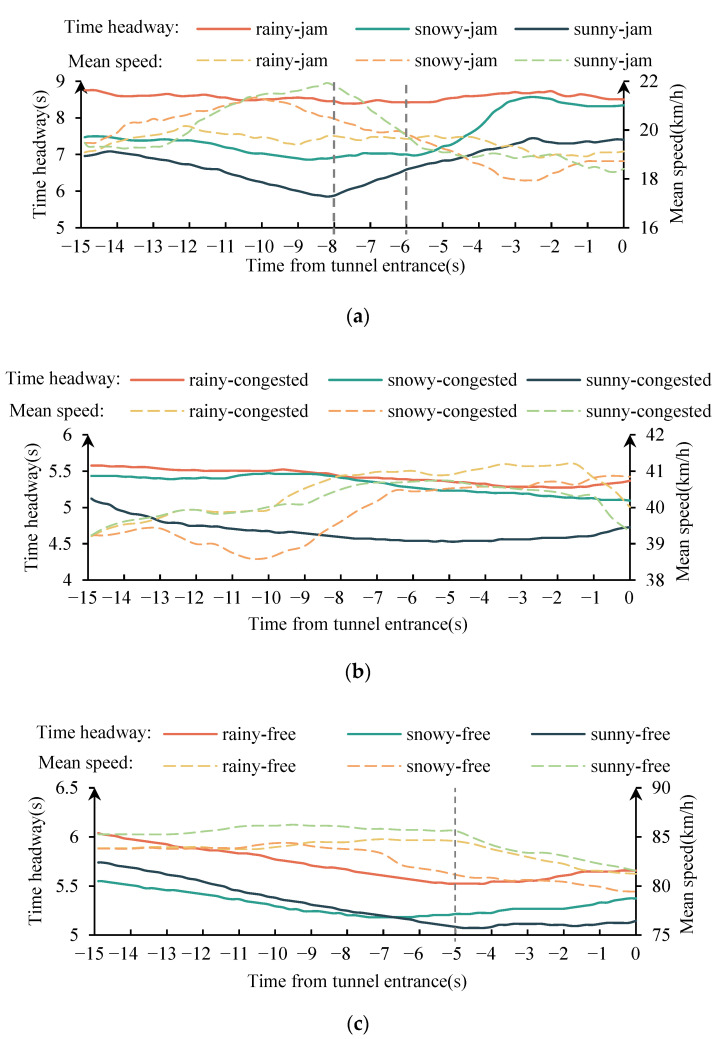
The mean change of the time headway within 15 s from the tunnel entrance. (**a**) jam flow condition. (**b**) congested flow condition. (**c**) free flow condition.

Figure 9 shows the mean change of the time headway within 15 s from the tunnel entrance under different traffic flow conditions in rainy, snowy, and fair weather. It can be seen that in the jam flow condition, the ranking of the average time headway in different weather is rainy weather > snowy weather > fair weather. In fair weather, the time headway increased at the position about 8 s away from the tunnel entrance, in snowy weather, the time headway increased at about 6 s away from the tunnel entrance, and in rainy weather, the time headway remained relatively constant. In the congested flow condition, the mean of time headway in different weather was ranked as rainy and snowy weather > sunny weather; the time headway was relatively constant in the whole car-following process. In the free flow condition, the mean of time headway in different weather was ranked as rainy weather > snowy weather and sunny weather. Prior to entering the tunnel entrance, the time headway decreased by about 15 s and increased by 5 s. The average time headway was greater than 4.5 s under all conditions, meeting the requirements of a comfortable time headway. Additionally, drivers increased the time headway when approaching the tunnel entrance, indicating they believed that traffic risk increased near the entrance and thus took a more cautious driving approach.

Additionally, this paper analyzed the influence of weather on the time headway under different traffic flow conditions. The value of the minimum time headway often represents the moment when the driver has the greatest risk during the process of car-following. The results of two-way ANOVA showed that traffic flow (*p* = 0.012 < 0.05) and weather (*p* = 0.061 < 0.1) had a significant impact on the minimum time headway, while the interaction between weather and traffic flow (*p* = 0.940 > 0.1) had no significant impact on the minimum time headway. Table 7 shows the significance test of time headway under different traffic conditions. Table 8 shows the significance test of time headway under different weather conditions.

**Figure 10:**
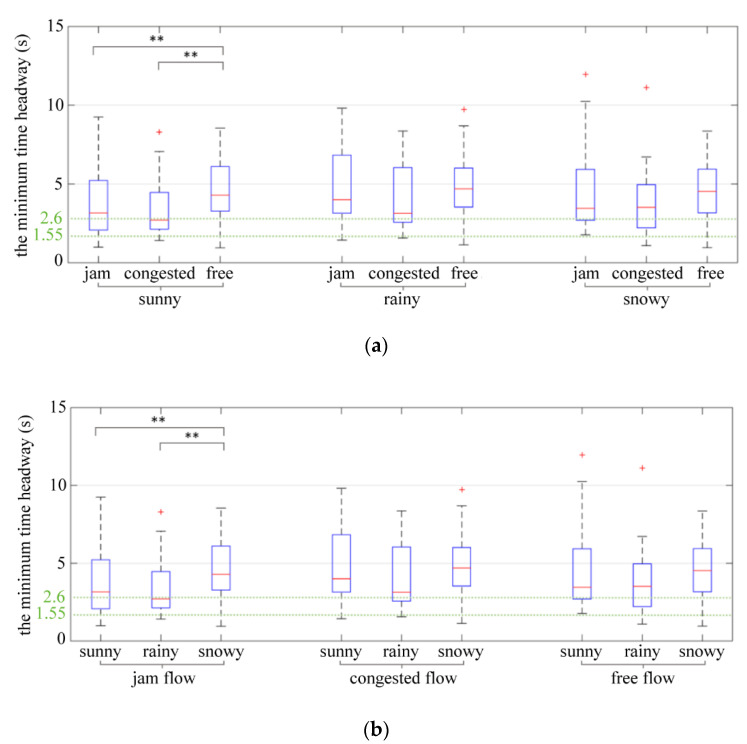
The minimum time headway. (**a**) The minimum time headway in different traffic flow conditions. (**b**) The minimum time headway in different weather conditions. ** indicates the variable was statistically significant at 0.05 level. + indicates outlier.

Another critical indicator of the stability of a car-following process is the standard deviation of time headway. The results of a two-way ANOVA showed that traffic flow (*p* = 0.000 < 0.05) had a significant impact on the standard deviation of time headway, while weather (*p* = 0.297 > 0.1) and the interaction between weather and traffic flow (*p* = 0.230 > 0.1) had no significant impact on the standard deviation of time headway. The results of further analysis of variance are shown in Table 9 and Figure 10.

**Figure 11:**
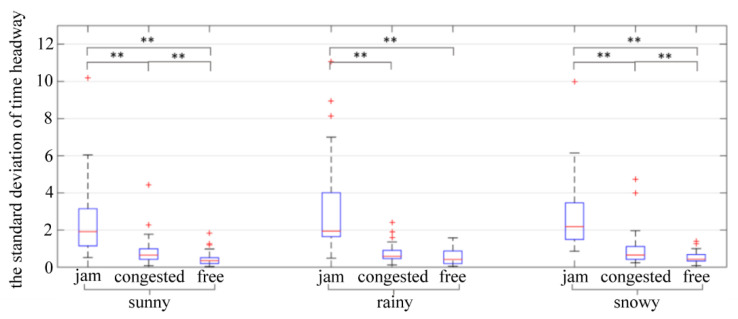
The standard deviation of time headway. ** indicates the variable was statistically significant at 0.05 level. + indicates outlier.

Based on the time headway characteristics of car-following behavior under different traffic flow conditions, it can be seen from Table 7 and Figure 10a that the minimum time headway in jam flow and congested flow conditions is smaller than that in the free flow condition, indicating that the driver of the following vehicle finds it easier to follow the vehicle ahead at a slower speed in congested flow and jam flow conditions. As the speed of the vehicle increases, the driver of the rear vehicle follows the vehicle ahead with a larger headway to ensure safety. It can be seen from Table 8 and Figure 11 that the mean of the standard deviation of the time headway under different weather conditions shows the law of jam flow > congested flow > free flow. When the vehicle speed is decreased, the dispersion of the time headway increases significantly, indicating that the driver tends to accelerate and decelerate frequently, which leads to poor longitudinal stability of the vehicle and a negative impact on traffic flow stability.

Comparing the time headway characteristics under various weather conditions, Table 10 and Figure 10b indicated that the weather had a weakly effect on time headway. In different traffic flow conditions, the minimum time headway in rainy and snowy weather was significantly higher than that in sunny weather. Specifically, rainy weather affected the minimum time headway in jam flow conditions, and the minimum time headway in rainy weather was significantly higher than in sunny weather. It indicated that the driver was more cautious in rainy and snowy weather.

### 3.3. The Influence of Different Conditions on Drivers’ Acceleration Behavior

**Figure 12:**
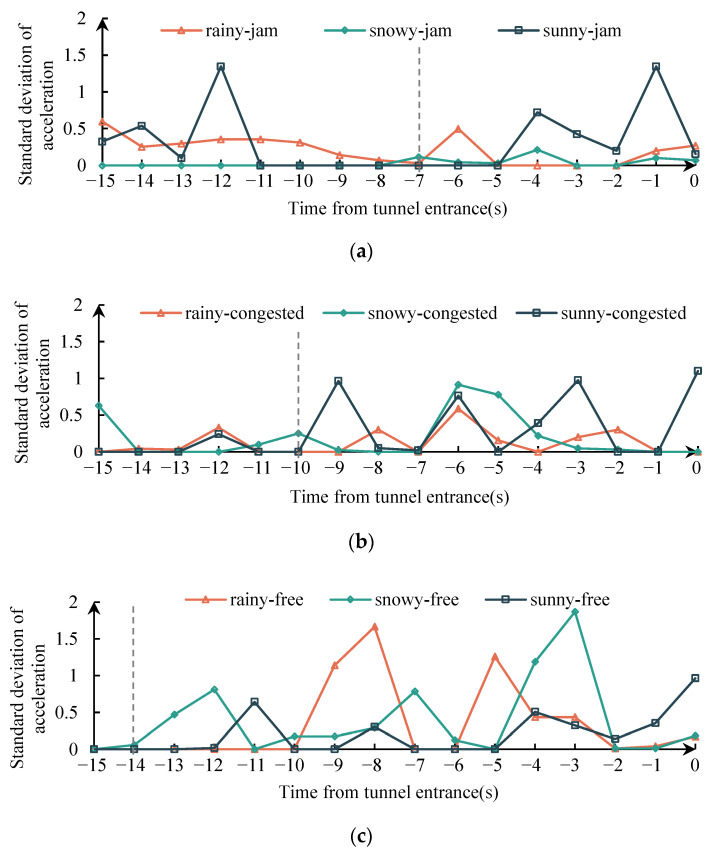
Standard deviation changes of average longitudinal acceleration within 15 s from the tunnel entrance. (**a**) jam flow. (**b**) congested flow. (**c**) free flow.

The driver took deceleration measures in response to the darkness of the tunnel entrance due to their driving experience, resulting in an increase in the mean of the standard deviation of longitudinal acceleration as the driver approached the tunnel entrance. Figure 12 illustrates the change in the standard deviation of the average longitudinal acceleration of the driver during the 15 s before tunnel entrance: In the jam flow condition, the mean of the standard deviation of acceleration increases significantly when it is approximately 7S away from the tunnel entrance; in the congested flow condition, the mean of the standard deviation of acceleration increases significantly when it is about 10 s away from the tunnel entrance; in the free flow condition, the mean of the standard deviation of acceleration increases significantly when it is approximately 14 s away from the tunnel mouth. It can be observed that as the speed of a vehicle increases, drivers tend to take deceleration measures as quickly as possible.

In order to explore the influence of weather and traffic flow on the standard deviation of acceleration during car-following, a two-way ANOVA was used to compare the mean. The results show that traffic flow conditions (*p* = 0.000 < 0.05) have a significant impact on the standard deviation of acceleration, while weather conditions (*p* = 0.294 > 0.1), and the interaction between weather and traffic flow (*p* = 0.377 > 0.1) have no significant impact on the standard deviation of acceleration. A further study was conducted on the influence of traffic flow on acceleration standard deviation. A Levene test was used to judge whether the variance meets the homogeneity requirement. The results are shown in Table 10.

**Figure 13:**
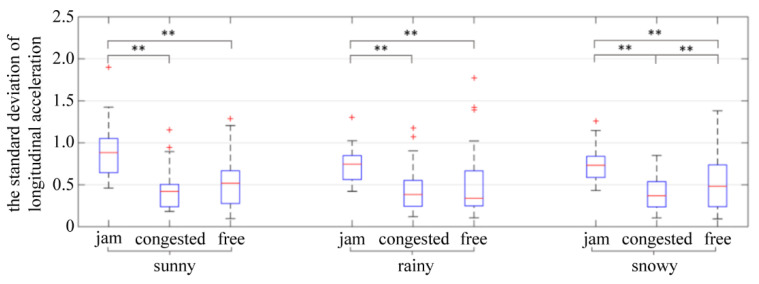
The standard deviation of longitudinal acceleration. ** indicates the variable was statistically significant at 0.05 level. + indicates outlier.

Figure 13 illustrates that, under different weather conditions, the standard deviation of longitudinal acceleration in jam flow is significantly greater than that in congested flow and free flow conditions. In the jam flow condition, the speed of the front vehicle was only maintained at 20 km/h, and the driver was forced to maintain a low speed to follow the car in front, which is much slower than expected. Therefore, the speed was increased as much as possible by frequent acceleration. However, after acceleration, the distance between the vehicle and the vehicle in front was shorter than the safety distance, so the driver frequently decelerated to increase the distance. In the jam flow condition, frequent acceleration and deceleration behavior caused a large standard deviation of longitudinal acceleration.

### 3.4. The Influence of Different Conditions on Lateral Offset

The standard deviation of lateral offset provides a reliable measure of the ability of the driver to drive on the centerline of the road. The greater the standard deviation of lateral offset, the worse the vehicle’s lateral stability and the more dangerous it becomes. In car-following, several drivers may veer left or right of the road centerline at the same time; therefore, the lateral offset may be positive or negative, similar to the acceleration index. The standard deviation can better reflect the violent fluctuations of the lateral offset than the average value. The two-way ANOVA showed that traffic flow (*p* = 0.000 < 0.05) had a significant impact on the standard deviation of lateral offset, while weather (*p* = 0.421 > 0.1), and the interaction between weather and traffic flow (*p* = 0.241 > 0.1) had no significant impact on the standard deviation of lateral offset. Table 11 shows the results of further ANOVA.

**Figure 14:**
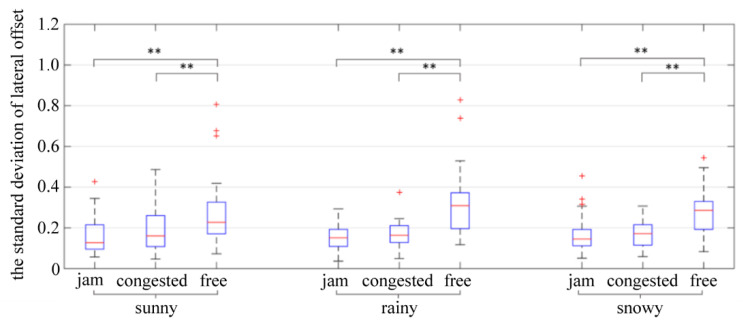
The standard deviation of lateral offset. ** indicates the variable was statistically significant at 0.05 level. + indicates outlier.

It can be seen from Figure 14 that the standard deviation of the lateral offset during car-following is greater in free flow conditions compared to jammed and congested flow conditions, indicating the driver’s lower ability to control the lateral stability of the vehicle in free flow.

The maximum lateral offset is the maximum distance the vehicle can deviate from the centerline of the road during a period of time. It can directly reflect the degree to which the vehicle deviates from the centerline of the road. It is an effective index to measure the lateral stability of the vehicle. The standard deviation of lateral offset can reflect the overall lateral offset of the vehicles, but it cannot reflect vehicles entering the lane edge. To determine whether the driver would affect the vehicles in the side lane during car-following at the tunnel entrance, the maximum lateral offset was used for analysis. The results of two-way ANOVA showed that traffic flow (*p* = 0.000 < 0.05) had a significant impact on the maximum lateral offset, while weather (*p* = 0.120 > 0.1), and the interaction between weather and traffic flow (*p* = 0.967 > 0.1) had no significant impact on the maximum lateral offset. Table 12 shows the results of further ANOVA.

**Figure 15:**
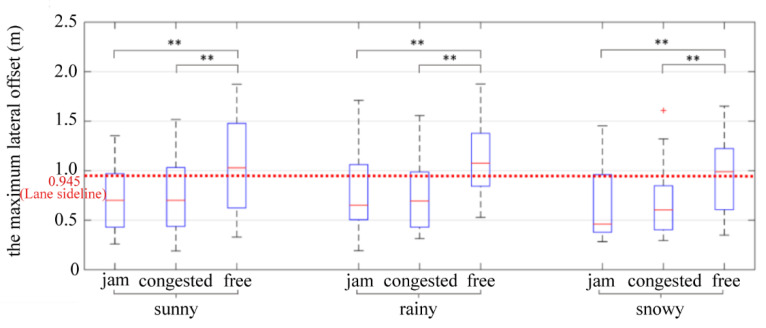
The maximum lateral offset. ** indicates the variable was statistically significant at 0.05 level. + indicates outlier.

Test results indicated that the maximum lateral offset in the free flow condition was significantly higher than that in jam flow and congested flow conditions, which was consistent with the standard deviation of lateral offset (see Figure 15). In the free flow condition, the driving speed was about 80 km/h, which was much faster than that of jam flow (20 km/h) and congested flow (40 km/h) conditions. In this case, assuming that the driving direction had the same offset angle, the lateral component of speed would also be faster in the free flow condition than in jam flow and congested flow conditions. The driver’s “cognition response” time was fairly constant, the maximum value and standard deviation of lateral offset were high in the free flow condition; lateral stability was the least stable compared to the other conditions.

### 3.5. The Influence of Different Conditions on Driver’s Emergency Response Time

**Figure 16:**
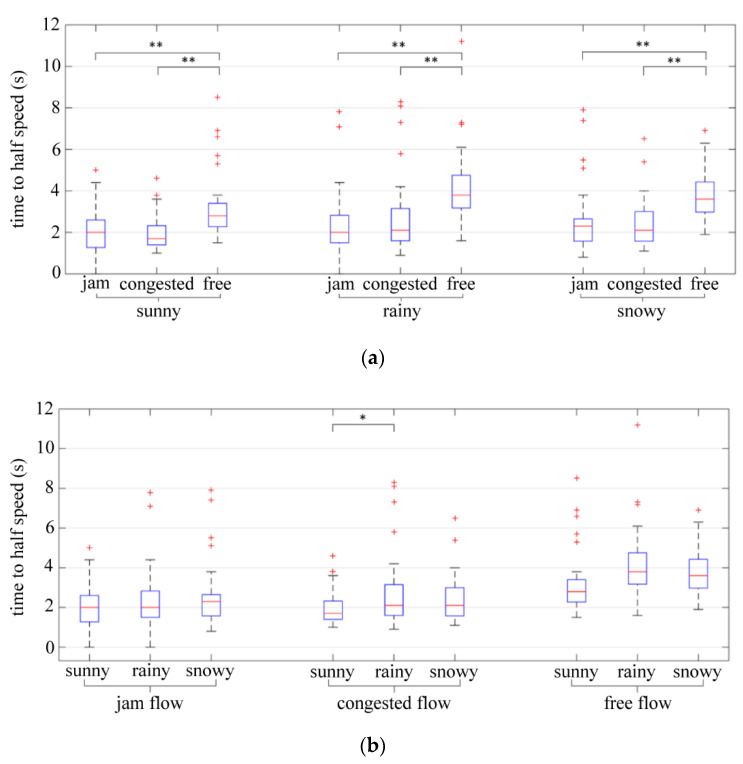
“Time to half speed”. (**a**) “Time to half speed” in different traffic flow conditions. (**b**) “Time to half speed” in different weather conditions. ** indicates the variable was statistically significant at 0.05 level. + indicates outlier. * indicates the variable was statistically significant at 0.1 level.

The two-way ANOVA was performed on the “time to half speed” collected in the experiment, and the results showed that weather (*p* = 0.003 < 0.05) and traffic flow (*p* = 0.00 < 0.05) all had significant effects on the mean of “time to half speed”, and the interaction of weather and traffic flow (*p* = 0.998 > 0.1) had no significant effect on this index. Furthermore, further analysis of the effect of weather and traffic flow conditions on “time to half speed” is presented in Table 13 and Table 14, and Figure 16.

As shown in Figure 16, in different weather conditions, “time to half speed” in the free flow condition is longer than that in the jam flow and congested flow conditions. The speed was fastest when the driver was traveling in free flow condition, so the process of slowing down would take more time than at other slower speeds. Two-way ANOVA results showed that weather had a significant effect on “time to half speed”, but further analysis results showed that only in the congested flow condition, the mean of “time to half speed” in rainy weather is significantly longer than that in sunny weather. The weather had a relatively weaker effect on the “time to half speed” than the effect of traffic flow.

### 3.6. The Influence of Different Slopes on Driving Behaviors

**Figure 17:**
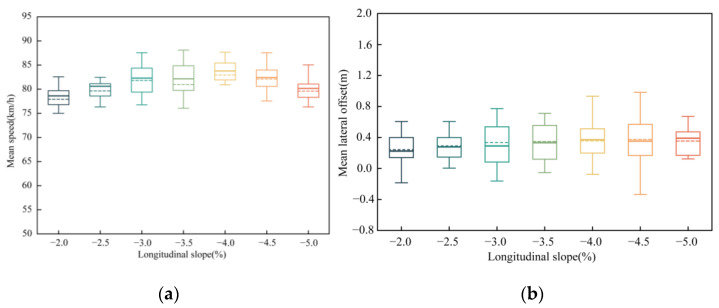
The relationship between driving behavior and longitudinal slope. (**a**) Mean speed. (**b**) Mean lateral offset.

Previous studies have shown that driving behavior in longitudinal segments of underground roads is greatly affected by the longitudinal slope. Moreover, longitudinal speed and lateral offset are the main indicators of driving behaviors [5,19,20]. Therefore, we will discuss the relationship between the driving behavior (longitudinal speed and lateral excursion are the main indicators of driving behavior) and the longitudinal slope of the cross-river and cross-sea tunnel entrance section. Seven sets of speed and lateral offset were obtained from seven different longitudinal slopes. The relationship between driving behavior and the longitudinal slope at the entrance of cross-river and cross-sea tunnel is shown in Figure 17.

As can be seen from Figure 17a, When the speed limit is 80 km/h and the longitudinal slope is 2.0% < i < 5.0%, the mean speed at the entrance of the cross-river and cross-sea tunnel is about 78~84 km/h. When the slope is less than about 4.0%, the speed increases as the slope increases. This is because when the slope is within the driver’s psychological safety range, drivers generally do not brake or brake slightly to drive safely to the bottom of the slope. Moreover, the speed increases with the slope due to the influence of gravity. However, when the slope is over 4.0%, the speed decreases as the slope increases. This is because the increase in slope gives the driver a sense of insecurity and makes the driver more cautious. This is because the increase in grade gives the driver a sense of insecurity and makes the driver more cautious. Drivers would increase the braking as the gradient increases to reduce the speed and avoid accidents. As shown in Figure 17b, vehicles tend to drive on the right, and the average fluctuation range of the lateral offset under different slopes is about 0.3 m. Different slopes have no significant effect on the offset of the driving trajectory.

Further analysis of variance was used to test whether the slope had a significant effect on speed and lateral offset. Sum of squares (SS), degree of freedom (df), mean square (MS), F value, and *p*-value are shown in Table 15. If *p* < 0.05, there is a significant relationship between the independent and dependent variables. As in the previous analysis, the slope of the cross-river and cross-sea tunnel has a significant effect on speed, but not on lateral offset.

## 4. Conclusions

This study aimed to understand the influence of traffic flow and weather conditions on the driver’s car-following behavior in terms of distance headway, time headway, longitudinal acceleration, lateral offset, and driver’s emergency response time on the entrance section of the cross-river and cross-sea tunnel. Based on the results from the driving simulator experiment, comparisons across scenarios were conducted, and the main findings were concluded below:

(1) On the entrance section of the cross-river and cross-sea tunnel, the driver’s car-following behavior is mainly affected by traffic flow conditions. Weather conditions mainly influence the time headway and driver’s emergency response time.

(2) On the entrance section of the cross-river and cross-sea tunnel, drivers tend to adopt more cautious driving behavior as the distance between vehicle and tunnel entrance decreases.

(3) There is a significant relationship between the slope of the cross-river and cross-sea tunnel entrance section and the driving speed. With the increase in the slope, the driver is more cautious, and the speed first increases and then decreases.

This study contains an analysis of the relationships between traffic flow, weather conditions, longitudinal slope, and driving behavior in the entrance section of a cross-river and cross-sea tunnel. This research provides a certain theoretical basis for cross-river and cross-sea tunnel traffic safety. However, the results are still in the basic research stage, further research seems necessary to discuss the interaction of traffic conditions and the slope of cross-river and cross-sea tunnels. A more focused experiment design on classifying the workload imposed by the tunnel environment is also needed.

## Figures and Tables

**Table 1:** Driving simulation scenarios.

	Traffic	Free Flow	Congested Flow	Jam Flow
Weather				
sunny weather		sunny-free	sunny-congested	sunny-jam
rainy weather		rainy-free	rainy-congested	rainy-jam
snowy weather		snowy-free	snowy-congested	snowy-jam

**Table 2:** Weather parameters set in SCANeR Studio^TM^.

Weather Condition	Rainfall Coefficient	Snowfall Coefficient	Adhesion Reduction Coefficient	
			Inside the Tunnel	Outside the Tunnel
sunny weather	-	-	1.00	1.00
rainy weather	0.6	-	1.00	0.60
snowy weather	-	0.45	1.00	0.45

**Table 3:** Results of traffic flow clustering based on speed.

Traffic Condition	Speed (km/h)	Number	Significance
congested	19.27	838	0.0068 **
jam	40.85	513	

** indicates the variable was statistically significant at 0.01 level.

**Table 4:** Variables Characterizing Driving Behaviors.

Classification	Variable	Unit
System Variable	Time	s
road variable	Road abscissa	m
	Road angle	°
	Road Id/Lane Id	-
	Lane type	-
	Intersection Id	-
vehicle variable	Speed/X/Y	km·h^−1^
	Acceleration/X/Y	m·s^−2^
	Lane gap/Road gap	m
	Yaw speed	(°)·s^−1^
	Yaw acceleration	(°)·s^−2^
	Car Spacing	m
	Speed Difference	km·h^−1^
Driver Operation Data	Steering at steering rack	°
	Steering wheel speed	(°)·s^−1^
	Gas pedal	-
	Brake pedal force	daN

**Table 5:** Significance test of the influence of traffic flow on the minimum distance headway.

Weather Condition	Variance Homogeneity Test	Mean Test		Post-Hoc		
		ANOVA	Welch ANOVA	Comparison Item	LSD	Games-Howell
sunny	0.000 **	-	0.000 **	Jam < Congested	-	0.005 **
				Congested < Free	-	0.000 **
				Jam < Free	-	0.000 **
rainy	0.000 **	-	0.000 **	Jam < Congested	-	0.008 **
				Congested < Free	-	0.000 **
				Jam < Free	-	0.000 **
snowy	0.000 **	-	0.000 **	Jam < Congested	-	0.022 **
				Congested < Free	-	0.000 **
				Jam < Free	-	0.000 **

** indicates the variable was statistically significant at 0.05 level.

**Table 6:** Significance test for the standard deviation of distance headways under different traffic flow conditions.

Weather Condition	Variance Homogeneity Test	Mean Test		Post-Hoc		
		ANOVA	Welch ANOVA	Comparison Item	LSD	Games-Howell
sunny	0.000 **	-	0.023 **	Jam < Congested	-	0.315
				Congested < Free	-	0.171
				Jam < Free	-	0.036 **
rainy	0.000 **	-	0.009 **	Jam < Congested	-	0.903
				Congested < Free	-	0.007 **
				Jam < Free	-	0.014 **
snowy	0.000 **	-	0.002 **	Jam < Congested	-	0.186
				Congested < Free	-	0.177
				Jam < Free	-	0.003 **

** indicates the variable was statistically significant at 0.05 level.

**Table 7:** The significance test of the influence of traffic flow conditions on the minimum time headway.

Weather Condition	Variance Homogeneity Test	Mean Test		Post-Hoc		
		ANOVA	Welch ANOVA	Comparison Item	LSD	Games-Howell
sunny	0.302	0.042 **	-	Jam < Congested	0.431	-
				Congested < Free	0.014 **	-
				Jam < Free	0.089 *	-
rainy	0.170	0.232	-	-	-	-
snowy	0.186	0.349	-	-	-	-

* indicates the variable was statistically significant at 0.1 level. ** indicates the variable was statistically significant at 0.05 level.

**Table 8:** The significance test of the influence of weather conditions on the minimum time headway.

Traffic Condition	Variance Homogeneity Test	Mean Test		Post-Hoc		
		ANOVA	Welch ANOVA	Comparison Item	LSD	Games-Howell
Jam	0.502	0.090 *	-	sunny < rainy	0.047 **	-
				snowy < rainy	0.355	-
				sunny < snowy	0.281	-
Congested	0.237	0.346	-	-	-	-
Free	0.966	0.712	-	-	-	-

* indicates the variable was statistically significant at 0.1 level. ** indicates the variable was statistically significant at 0.05 level.

**Table 9:** The significance test of the influence of traffic flow conditions on the standard deviation of time headway.

Weather Condition	Variance Homogeneity Test	Mean Test		Post-Hoc		
		ANOVA	Welch ANOVA	Comparison Item	LSD	Games-Howell
sunny	0.000 **	-	0.000 **	Jam < Congested	-	0.000 **
				Congested < Free		0.035 **
				Jam < Free		0.000 **
rainy	0.000 **	-	0.000 **	Jam < Congested	-	0.000 **
				Congested < Free		0.233
				Jam < Free		0.000 **
snowy	0.000 **	-	0.000 **	Jam < Congested	-	0.000 **
				Congested < Free		0.031 **
				Jam < Free		0.000 **

** indicates the variable was statistically significant at 0.05 level.

**Table 10:** The significance test of the effect of traffic flow on the standard deviation of acceleration.

Weather Condition	Variance Homogeneity Test	Mean Test		Post-Hoc		
		ANOVA	Welch ANOVA	Comparison Item	LSD	Games-Howell
sunny	0.171	0.000 **	-	Jam < Congested	0.000 **	-
				Congested < Free	0.351	-
				Jam < Free	0.000 **	-
rainy	0.004 **	-	0.000 **	Jam < Congested	-	0.000 **
				Congested < Free	-	0.778
				Jam < Free	-	0.030 **
snowy	0.001 **	-	0.000 **	Jam < Congested	-	0.000 **
				Congested < Free	-	0.027 **
				Jam < Free	-	0.004 **

** indicates the variable was statistically significant at 0.05 level.

**Table 11:** The significance test of the effect of traffic flow on the standard deviation of lateral offset.

Weather Condition	Variance Homogeneity Test	Mean Test		Post-Hoc		
		ANOVA	Welch ANOVA	Comparison Item	LSD	Games-Howell
sunny	0.019 **	-	0.006 **	Jam < Congested	-	0.612
				Congested < Free	-	0.036 **
				Jam < Free	-	0.004 **
rainy	0.001 **	-	0.000 **	Jam < Congested	-	0.683
				Congested < Free	-	0.000 **
				Jam < Free	-	0.000 **
snowy	0.294	0.000 **	-	Jam < Congested	0.862	-
				Congested < Free	0.000 **	-
				Jam < Free	0.000 **	-

** indicates the variable was statistically significant at 0.05 level.

**Table 12:** Significance test of the effect of traffic flow on the maximum lateral offset.

Weather Condition	Variance Homogeneity Test	Mean Test		Post-Hoc		
		ANOVA	Welch ANOVA	Comparison Item	LSD	Games-Howell
sunny	0.036 **	-	0.002 **	Jam < Congested	-	0.913
				Congested <Free	-	0.008 **
				Jam < Free	-	0.002 **
rainy	0.988	0.000 **	-	Jam < Congested	0.980	-
				Congested <Free	0.000 **	-
				Jam < Free	0.000 **	-
snowy	0.443	0.001 **	-	Jam < Congested	0.663	-
				Congested <Free	0.002 **	-
				Jam < Free	0.001 **	-

** indicates the variable was statistically significant at 0.05 level.

**Table 13:** Significance test of the influence of traffic flow on “time to half speed”.

Weather Condition	Variance Homogeneity Test	Mean Test		Post-Hoc		
		ANOVA	Welch ANOVA	Comparison Item	LSD	Games-Howell
sunny	0.015 **	-	0.000 **	Jam < Congested	-	0.961
				Congested < Free	-	0.000 **
				Jam < Free	-	0.001 **
rainy	0.822	0.009 **	-	Jam < Congested	0.930	-
				Congested < Free	0.009 **	-
				Jam < Free	0.007 **	-
snowy	0.658	0.000 **	-	Jam < Congested	0.713	-
				Congested < Free	0.000 **	-
				Jam < Free	0.000 **	-

** indicates the variable was statistically significant at 0.05 level.

**Table 14:** Significance test of the influence of weather on the time taken to reduce to half speed.

Traffic Condition	Variance Homogeneity Test	Mean Test		Post-Hoc		
		ANOVA	Welch ANOVA	Comparison Item	LSD	Games-Howell
Jam	0.133	0.250	-	-	-	-
Congested	0.012 **	-	0.031 **	sunny < rainy	-	0.070 *
				snowy < rainy	-	0.699
				sunny < snowy	-	0.124
Free	0.768	0.117	-	-	-	-

* indicates the variable was statistically significant at 0.1 level. ** indicates the variable was statistically significant at 0.05 level.

**Table 15:** Variance analysis of the relationship between driving behavior and slope.

Variables		SS	df	MS	F Value	*p* Value
Speed	Variance between groups	580.186	6	96.697	8.889	1.198 × 10^−8^
	Intraclass variance	2284.424	210	10.878		
Lateral offset	Variance between groups	0.744	6	0.124	1.77	0.106
	Intraclass variance	14.695	210	0.069		

## Data Availability

The data that support the findings of this study are available from the corresponding author, upon reasonable request.
